# Home-Based Care, the Missing Link in Caring of Patients Living with HIV/AIDS and Their Family Members: A Narrative Review

**DOI:** 10.30476/ijcbnm.2020.82771.1085

**Published:** 2020-07

**Authors:** Mona Larki, Robab Latifnejad Roudsari

**Affiliations:** 1 Student Research Committee, School of Nursing and Midwifery, Mashhad University of Medical Sciences, Mashhad, Iran; 2 Nursing and Midwifery Care Research Center, Mashhad University of Medical Sciences, Mashhad, Iran; 3 Department of Midwifery, School of Nursing and Midwifery, Mashhad University of Medical Sciences, Mashhad, Iran

**Keywords:** Acquired immunodeficiency syndrome, Healthcare systems, home-based health care, home-care services, Human immunodeficiency virus

## Abstract

Inconsistencies between the number of patients, qualified caregivers and lack of adequate services and resources in the healthcare systems for people living with HIV/AIDS have led to the idea of providing healthcare services for this vulnerable population at home. This study aimed to review the evidence related to the Home-Based Care (HBC) programs in the context of HIV. Literature search was carried out without publication date limit through English databases of Cochrane Library, PubMed, EBSCO, Scopus, Google Scholar, Science Direct, as well as Persian databases including Magiran and SID by the end of July 2019. Out of 1312 studies retrieved from the search of databases, six guidelines and 50 articles met the eligible criteria. The results showed that the overall purpose of HBC is to create hope through providing appropriate care to help the patients and their families to maintain their independence and have the best possible quality of life. The potential benefits of HBC could be discussed in three main categories: 1) patients, including patients’ satisfaction, quality of life, adherence to antiretroviral therapy, preventing mother-to-child transmission, as well as biochemical, social and psychological outcomes, 2) families, consisted of promotinon of family members’ participation, enhancement of family members’ awareness and provision of voluntary counseling and testing, and 3) healthcare systems, containing health care costs, workload at healthcare centers and time spent on services. Given the lessons learnt from the existing HBC programs, developing an alternative approach for providing healthcare at home in the context of HIV seems necessary and could be an agenda for action in health policy making in Iran.

## INTRODUCTION

AIDS is the fourth leading cause of death now and will become the first cause of death by 2020 in the world. ^1^ It is the second most common cause of mortality among communicable diseases. In 2017, it was estimated that 37.3 million people lived with HIV(PLWH) worldwide. ^2^ Iran with an estimated 66000 PLWH and due to its location in the Middle East region is at risk of AIDS. ^3^ It is anticipated that the highest growth rate of illness burden in Iran are related to the growth of HIV/AIDS by 2025. ^4^

Today, by changing the health care systems, most patients receive professional and specialized care at home after discharge from the hospital. ^5^ Home-Based Care (HBC) is sensitive to the culture and value system of the local community- a sensitivity that is often missing in clinical hospital settings. ^6^ HBC is defined by the Committee on the National Strategy for AIDS (CNSA) as “care at the patient’s place of residence to complement or replace hospital care services including medical management, palliative care and social support”. ^7^ HBC is a common approach to taking care of PLWH outside of the health facility environment, especially in epidemic areas ^8^ and under the umbrella of primary health care. ^9^

More recently, the model of care for PLWH has also shifted from hospital-based care to HBC, due to the increasing number of patients, inconsistencies between the number of patients and qualified health care providers, lack of space, and increasing hospital costs. ^10^ Additionally, long-term hospitalization, as well as not responding to the patients’ needs, is always considered as a challenge in the care of PLWH. ^10 , 11^ The research has identified additional barriers to care services access and delays in seeking appropriate healthcare, in PLWH, such as: sadness, depression and, isolation. ^12^

Hence, HBC has been proposed as part of a new, integrated and flexible approach to care, management, support and treatment of PLWH as well as their families. ^13 - 15^ HBC focuses on family solidarity. It also emphasizes a strong basic principle on preserving the traditional care patterns, which encourages family members like the spouse, parents and friends to take key responsibility in caring of their patients. In this process, the family is assisted in accepting the conditions of the patient and providing him with the necessary care and support such as adherence to the medical and nutritional requirements. ^16^

This type of care can be considered as an appropriate and consistent tool for achieving UNAIDS goals for HIV/AIDS treatment coverage. ^17^ HBC programs officially began in Iran about 20 years ago, but its dimensions has remained unknown. ^18^ The results of the research conducted in Iran revealed that the health care delivery systems have the physician- and hospital- based management; so it is essential that policymakers reform the health systems towards community based management. ^19^ Based on the recent studies in Iran, HBC has confronted numerous challenges including policy deficiency, lack of thorough guidelines, financial problems, professional barriers, bureaucratic tensions, and lack of familiarity with this type of care in the community. ^18 , 20^

Despite the considerable development on HBC, it is a missing link in caring the patients living with HIV/AIDS and their family members across the world in general and in Iran in particular. Also, there is little information about it in the context of HIV. The current review aimed to elaborate the definition, history, objectives, components and principles, models of care, effectiveness and challenges of HBC programs in order to provide a new insight based on the updated global evidence related to HIV/AIDS.

## MATERIALS AND METHODS

This narrative review was conducted to get a comprehensive perspective of HBC in HIV/AIDS patients. The databases searched for relevant articles included English databases of Cochrane Library, PubMed, EBSCO, Scopus, Science Direct, Google Scholar, as well as Persian databases including Magiran and SID. Keywords for search were: ‘HIV/AIDS infection’, ‘home-based care’, ‘home-care services’, ‘community health workers and HIV’, ‘caregiving and HIV’ ‘HIV care’ and ‘treatment’ as well as ‘community health services’. To increase precision in the search process, we used Boolean terms (AND/OR) to separate the keywords as well as medical subject headings (MeSH). Additionally, relevant guidelines about home-based care and community-based care in HIV/AIDS were searched in databases of National Guideline Clearinghouse (NGC), SIGN clinical guidelines and, Google Scholar. These guidelines included: National Guidelines for Community and Home Based Care, ^12^ Community home-based care in resource-limited settings, ^21^ National guidelines for the clinical management of HIV and AIDS, ^22^ HIV/AIDS Home-Based Care Costing Guidelines, ^23^ Reducing HIV stigma and discrimination: a critical part of national AIDS programs, ^24^ and planning, implementing and monitoring home-based HIV. ^25^

Year of publication and type of study design were not restricted in order to obtain all the literature on HBC in HIV. The search was performed by the end of 27th of July 2019. 

Secondary searching was also conducted through grey literature such as reports of World Health Organization (WHO), UNAIDS, The United Nations Educational, Scientific and Cultural Organization (UNESCO) websites; unpublished manuscripts; and dissertations reporting evidence on home-based care. Reference lists of the included studies were also reviewed to find additional references. 

### *Inclusion Criteria for Studies*

1. Studies providing the evidence regarding individuals with HIV/AIDS engaged in HBC 

2. Studies reporting the evidence of using HBC as an intervention in patient’s family members

3. Guidelines related to the home-based care in the context of HIV/AIDS

4. Original research articles using qualitative or quantitative approaches

### *Exclusion criteria*

Articles were excluded if:

1. Their language was not English or Persian 

2. There was no full text 

3. It was published as a letter to editor or conference abstract

**Figure 1:**
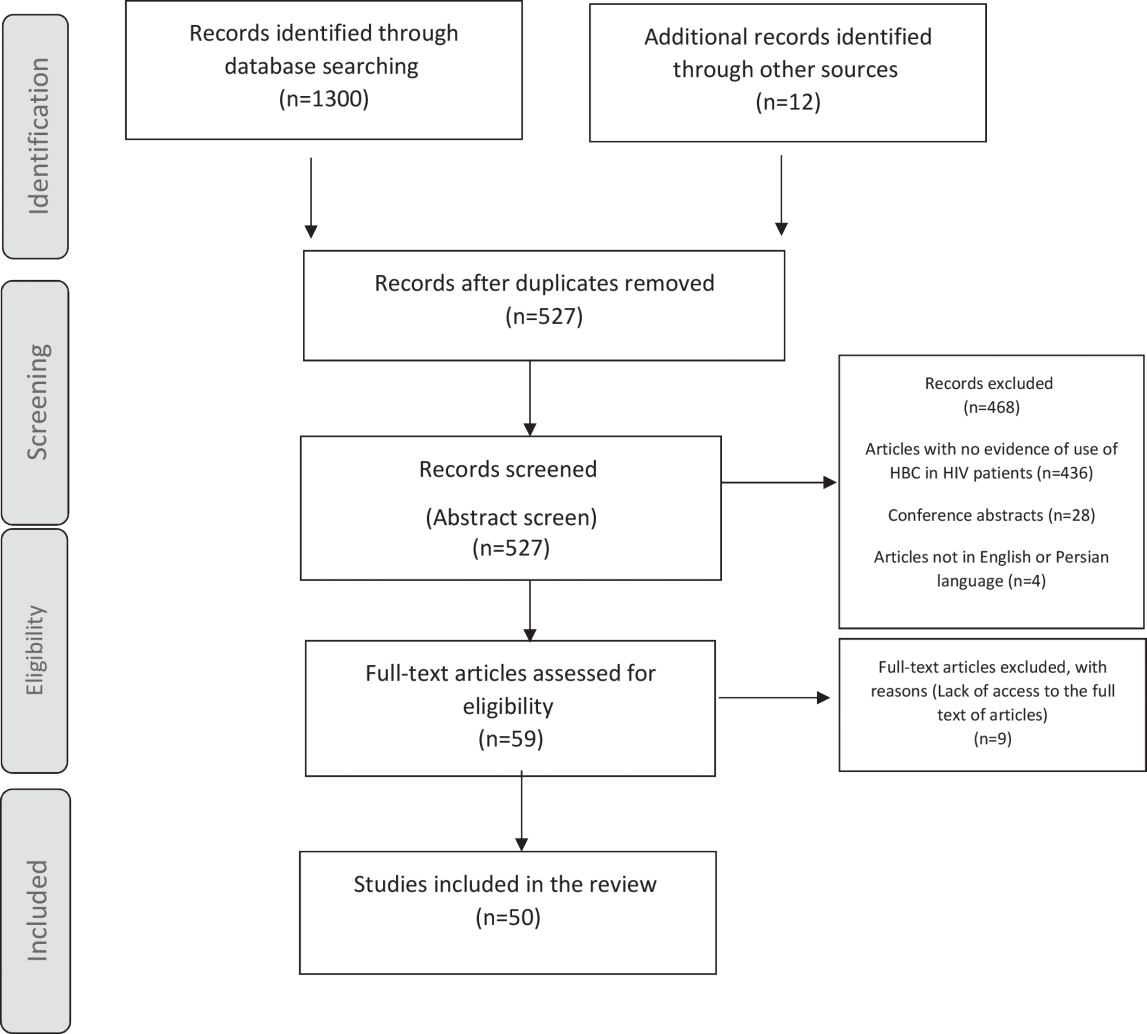
PRISMA ﬂow diagram

The strategy for selecting studies consisted of four steps: identifying all related literature; screening of abstract for eligible and exclusion criteria; assessing eligibility of the full text; and finally including selected studies in the review process. ^26^ See Figure 1 for the number of records retrieved and included in each of these steps. It should be noted that ethical issues such as avoiding from plagiarism, keeping robustness in data extraction, data preparation and submission were observed by the authors. We made an attempt to categorize the collected information and present a comprehensive explanation and interpretation of the challenges, and the facilities needed for HBC establishment in Iran. The results of the study are in accordance with the Preferred Reporting Items for Systematic Reviews and MetaAnalyses (PRISMA) guideline. ^26^

## RESULTS

In the initial phase of the study, 1312 articles were identified, from which 785 articles were duplicated and excluded. A total of 468 were also omitted after screening of the abstracts. Full-text screening resulted in exclusion of an additional 9 articles due to the lack of access to the full text of the articles. Finally, a total of 50 relevant studies remained for data extraction. In addition, six retrieved guidelines were also included in the review. Findings obtained from 50 articles and six guidelines were categorized into sections of definition, history, objectives, principles and components, types of care models and benefits of HBC in relation to the HIV/AIDS. 

### *Characteristics of the Included Studies*

Of the included studies about effects of home based care, 17 were conducted in Uganda, ^27 - 43^ 11 in USA, ^44 - 54^ three in Kenya, ^55 - 57^ four in the UK, ^58 - 61^ two in China, ^62 , 63^ South Africa, ^64 , 65^ and Zambia, ^66 , 67^ and one in Mozambique, ^68^ Tanzania, ^6^ 9 Vietnam, ^70^ Pakistan, ^71^ Italy, ^72^ Norway, ^73^ Nepal, ^74^ Namibia, ^75^ and Netherland, ^44^ All the included studies were published during the years 1991 and 2019. The total number of participants in all included studies was 29092. Of the included studies, six were qualitative, ^58 , 64 , 67 , 73 , 75 , 76^ 19 were randomized control trial, ^34 , 38 , 44 , 46 - 56 , 62 , 63 , 66 , 69 , 74^ four were cross-sectional studies, ^40 , 45 , 57 , 71^ and 17 were cohort studies; ^27 - 30 , 32 , 33 , 35 , 37 , 39 , 41 - 43 , 59 , 60 , 65 , 68 , 72^ Finally, there was one case control, ^40^ one cost-effective, ^36^ one mixed method, ^31^ and one pilot descriptive study. ^61^

Data from six guidelines were also extracted to elaborate the definition, ^12^ history, ^12 , 21^ objectives, ^12 , 21 , 22^ component and principles, ^12 , 22 - 24^ models of care, ^21 , 25^ and challenges. ^12 , 22 , 23^

### *1. Definition of HBC*

According to the World Health Organization, home- based care is a kind of care for PLWH and their affected families in their homes. It includes physical, psychosocial, palliative and spiritual care, clinical surveillance and opportunistic infections’ management (prevention and treatment), counseling, nutrition supplements, and healthy water intake. ^12^

### *2. The History of HBC in HIV/AIDS*

A review of studies suggests that home-based care programs for PLWH mainly started in North America and Europe in the late 1980s. ^12^ At the beginning of these programs in the United States, the National AIDS Strategy (1986) stated that HBC must be comprehensive, cost-effective, accurate and, carefully coordinated and monitored. Also, hospitalization should only be done if necessary. In sub-Saharan Africa and other developing countries, home-based care programs were developed as non-systemic and need-based interventions, when other care options seemed necessary to confront with the effects of HIV / AIDS. ^21^ The initiation of this care was provided by non-governmental organizations (NGO’s), faith-based organizations (FBO’s), and community-based organizations (CBO’s). Also, it was provided by a set of staff, including volunteers, community health workers, nurses, doctors and other professionals. ^12^

### *3. The Objectives of HBC in HIV/AIDS*

These interventions are usually developed with a clear purpose, which is dependent on the population, cultural context, epidemic type, geographic location, political environment, budget and resources available. ^25^ The overall purpose of these programs is to create hope through providing quality and appropriate care to help patients and their families to maintain their independence and obtain the best possible quality of life. ^21^

The Specific Objectives of HBC in PLWH Include:

1. To ensure that patients receive physical specific care and treatment.

2. To ensure the effectiveness of opportunistic infections and antiretroviral treatment. 

3. To ensure that patients receive mental health and social counseling.

4. To ensure that patients and their families benefit from community-based social networking support.

5. To develop cooperation relationships between public health and professional networks.

6. To raise community awareness on AIDS prevention and care and support needs of patients.

7. To Provide palliative care at the end of life. ^12 , 22^

### *4. Principles and components*

**Principles of HBC in PLWH:** According to a UNAIDS study in 2007, HBC for PLWH includes seven categories of activities: 

**Providing proper care:** to provide comprehensive primary care; advanced tuberculosis care; improved screening and treatment of sexually transmitted infections.

**Continuum of care:** to provide HIV prevention training, home-based care, treatment and support for PLWH.

**Training:** to train community leaders on HIV counseling and home-based care conducted by peer-education training programs to achieve behavioral changes.

**Supplies and equipment:** to initiate discussions among hospital managers and staff about the service received by PLWH.

**Necessary human resources:** to provide PLWH with support and resources to tackle internalized stigma and to engage in advocacy.

**Sustainable financing:** to provide home-care assistance and financial and spiritual support for PLWH at home.

**Monitoring and evaluation:** it should be key elements of HIV programming. ^24^

Table 1 describes the components of HBC in accordance with guidelines. ^12 , 22 , 23^

**Table 1:** Principles and components of Home Based Care in HIV/AIDS

Principles	Components
Counseling and testing^12, 22^	Counseling at home
	Doing home tests
	Referring at home to health centers for testing HIV
Antiretroviral therapy^12, 22^	Changes in antiretroviral therapy
	Consultation on adherence and monitoring of drugs
	Referral due to side effects and adverse effects of medicines
Preventing Mother-to-Child Transmission of HIV^12^	Prophylaxis treatment for mother and child
	Counseling about breastfeeding and nutrition for mothers
	Family planning services
	Involvement of men in mother to child transmission prevention programs
	Diagnosis and referral of the child for treatment at the beginning of childhood
	Infection control
Palliative care^23^	Pain management and symptom control using NSAIDs, codeine and other opioids
	Spiritual support
Tuberculosis / HIV care^22^	Evaluation of TB in patients
	Treatment of opportunistic infections
	Infection control
Nutrition^12, 23^	Nutrition education
	Nutrition and alternative therapies
	Appropriate food plans with medicinal diet
Psychosocial and legal support^12^	Communication between supporter groups
	Overcoming fear and concern in patients
	Maintaining confidentiality of patient information
	Observing the patients’ rights and their desires in receiving services
Sexually Transmitted Infections^12, 22^	Identification, treatment and referral of cases
	Informing the sex partner
	Infection control
Education-Information and Communications^12, 22^	Health education for family members (skin, hair, oral care and environmental care)
	Positive prevention
	ABCD approach: Abstinence, Being faithful, Condom promotion and diagnostic HIV testing
	Health promotion
	Infection control
Qualified staff^22^	Adequate training
	Appropriate payment to them

### *5. HBC Models in HIV/AIDS*

The World Health Organization states that there are basically two kinds of HBC: formal and informal. Formal care begins and runs through an official structure. While informal care is provided by family members, who are the first provider of care. Sometimes, this kind of care is done by the friends of affected people without payment cost. ^21 , 25^ Table 2 describes the models of HBC in HIV/AIDS.

**Table 2:** Home-based care Models in HIV/AIDS

Models	Object	Outcome
Door-to-Door^25^	Covering a high level of service in a particular community or geographic location	Faster diagnosis of patients
		Increased disclosure among the couples, member families
		Help to cover the disabled and marginalized groups
Index-Patient Model^25^	Providers of services refer to homes for people with HIV or TB, and provide services to their partner and other members of the family with their consent	Identification of serodiscordant couples in high-risk groups,
		Provision of conditions for disclosing their status along with risk reduction education
		Family planning
		Safer pregnancy counseling
		Promotion of condom use
Self-Testing Model^21^	Provision of services for health care workers and their partners	Improvement of accessibility, convenience and confidentiality of test results

### *6. The Benefits of HBC*

An overview of the studies reveals the significant benefits of this approach in patients, families and the community. These benefits are summarized in three categories of benefits for the patients, families and healthcare systems. Detailed information on the design, participants, setting, and outcomes of these studies can be found in Table 3.

**Table 3:** Characteristics of studies included in the review

Author and Date	Participants	Country	Design	Findings
McCann 1991^58^	Gay men positive for HIV. N=265	UnitedKingdom	Qualitative	Increased patients’ satisfaction in related to reassurance and support
Tramarin 1992^72^	PLWH^a^ accessing home care N=10 and hospital care N=32	Italy	Prospective cohort-follow up 6 months	Lower costs for patients with advanced disease accessing home- based care
Butters 1992^59^	PLWH from 2 centers. N=140	UnitedKingdom	Prospective cohort- follow up 2 weeks	Significant improvements in pain symptoms control and anxiety in patients
Moons 1994^76^	PLWH. N=13	Netherlands	Qualitative-exploratory	Patients received psychological support and high quality of care
Butters 1995^60^	PLWH from multicenter. N=234	UnitedKingdom	Prospective cohort- follow up 6 weeks	Significant changes in worsening symptom control, improving spiritual status, patient/ family communication and patient insight
Foley 1995^45^	PLWH. N=50	United States of America	Cross sectional	Clients were very satisfied with AHOP (At Home Options Program) services.
Koffman 1996^61^	PLWH with advanced HIV/AIDS. N=36	UnitedKingdom	Descriptive pilot	Significance improvement in symptom control and family insight
Nickel 1996^46^	Home care PLWH. N=57	United States of America	RCT^b^- follow up 6 months	No signiﬁcant differences in the quality of life scores or the survival curves
Cherin 1998^47^7	PLWH. N=549	United States of America	RCT- follow up 20 months	No signiﬁcant difference in average length of time on service
Flatley-Brennan 1998^48^	PLWH. N=57	United States of America	RCT- follow up 6 months	Reduction in social isolation in the case of control of depression
Bunch 1998^73^	PLWH. N=64	Norway	Qualitative thematic analysis	The cost was less than traditional hospitalization and no more medical complications
Gustafson 1999^49^	PLWH. N=204	United States of America	RCT- follow up 6 months	Improved a patient’s quality of life. Reduced the time spent with providers
Uys 2001^64^	Professionals working in HIV. N=36	South Africa	Qualitative	HBC is cost-effective and provides social support
Wang 2010^62^	PLWH. N=116	China	RCT- follow up 10 months	Improved self-reported adherence, WHO quality of life measures and reduced symptoms of depression.
Miles 2003^50^	PLWH. N=109	United States of America	RCT- follow up 6 months	No difference in depressive symptoms, mood, general health or overall functioning (Health related quality of life). Signiﬁcant difference in HIV stigma
Fylkesnes 2004^66^	Household members of patients. N=2445	Zambia	RCT- follow up 3 yeas	High acceptability of VCT^c^
Berrien 2004^51^	HIV-positive children. N=37	United States of America	RCT- with follow up 12 months	Adherence improved. No signiﬁcant differences in CD4 counts or viral loads.
Mermin 2004^27^	509 PLWH, 1522 negative household members	Uganda	Prospective cohort -follow up 5 months	Increased CD4-cell count and decreased viral load.
Bunuel 2006^42^	PLWH. N=926	Uganda	Prospective cohort- follow-up 6 months	Significant decreased median viral load
Weidle 2006^41^	PLWH with advanced HIV. N=987	Uganda	Prospective cohort - follow up 12 months	Decrease in viral loud and good adherence to antiretroviral therapy
Were 2006^40^	Household member of patients. N=2373	Uganda	Cross sectional	Detection of a large number of previously undiagnosed HIV infections and HIV-discordant relationships
Dolan 2006^52^	PLWH. N=40	United States of America	RCT- follow up 16 weeks	No significant difference was seen in lipid levels, blood pressure, or abdominal visceral fat.
Williams 2006^53^	PLWH. N=171	United States of America	RCT- follow up 15 months	No signiﬁcant differences in CD4 counts or viral loads.
Apondi 2007^39^	PLWH. N=654	Uganda	Prospective cohort- follow up 3 months	Positive social outcomes including family or community support and relationship strengthening and reduced stigma
Amuron 2007^38^	PLWH. N=1453	Uganda	RCT- follow-up 36 months	Decreased plasma viral load
Mermin 2008^37^	466 PLWH and 1481 negative household members	Uganda	Prospective cohort - follow up 5 months	Reduced mortality and orphan hood among adults with HIV and their families
Marseille 2009^36^	PLWH. N=1045	Uganda	Cost effectiveness	More cost-effective than estimates for facility-based ART^d^
More cost-effective than estimates for facility-based ARTdJaffar 2009^34^	PLWH. N=1453	Uganda	RCT- follow up 36 months	Significant decreased plasma viral load. Costs were similar for facility-based ART^da^
Wools-Kaloustian 2009^55^	PLWH. N=208	Kenya	RCT- follow up 2 years	Stigma-related events decreased
Wouters 2009^65^	PLWH. N=268	South Africa	Prospective Cohort - follow up 36 months	Increased adherence to ART^4^; improved disclosure to family members
Kipp 2010^33^	PLWH. N=385	Uganda	Prospective cohort- follow up 6 months	No significant virologic suppression
Selke 2010^56^	PLWH. N=189	Kenya	RCTb- follow up 1 year	No significant detectable viral load, mean CD4 count
Wanyenze 2010^35^	PLWH. N=689	Uganda	Cohort- follow up 8 years	Waiting time was the longest at HBCe compared with clinic-based care
Alibhai 2010^32^	PLWH. N=130	Uganda	Prospective cohort- follow up 1 year	Significant improvements in health-related quality of life
Mermin 2011^28^	PLWH. N=1094	Uganda	Prospective cohort - follow up 3 years	Decreased viral loud and increased CD4
Amuron 2011^43^	PLWH. N=1453	Uganda	Prospective cohort- follow-up 36 months	Mortality rates were less in the home care than facility-based arm.
Kipp 2011^29^	PLWH. N=385	Uganda	Prospective cohort- follow-up 6 months	Achievement of viral suppression- good adherence to treatment and slightly more cost-effective. Costs were similar for facility-based ART^d^d
Decroo 2011^68^	PLWH. N=1301	Mozambique	Retrospective cohort -12.9 months	Improved access. Highly satisfactory care
Hanrahan 2011^54^	PLWH. N=238	United States of America	RCT- with follow up 12 months	No clear difference in health-related quality of life outcomes (SF-12^f^)- No significant difference in reduction in psychiatric symptoms
Arem 2011^31^	Patients as peer health workers. N=29	Uganda	Mixed method	Reduced stigma; reduced workload
Oguntibeju 2011^75^	PLWH. N=31	Namibia	Qualitative study-thematic analysis	Access the HBC^e^ services, reduced stigma associated with HIV infection
Kipp 2012^30^	PLWH. N=385	Uganda	Prospective cohort- follow up two years	Successful ART^d^ treatment outcomes in the community-based cohort were equivalent to those in the hospital-based cohort.
Williams 2014^63^	PLWH. N=110	China	RCT- with follow up 12 months	Improved self-reported adherence. No significant difference in social support and stigma, differences in CD4 counts or viral loads.
Kohler 2014^57^	Random sample. N=405 women with HIV. N=247	Kenya	Cross sectional	Provision of PMTCT^g^ education and further reductions in vertical transmission
Blank 2014^44^	HIV-positive adults. N=238.	United States of America	RCT- follow up 24 months	SF-12^f^ mental health subscale improved but the SF-12 physical health subscale did not
Cataldo 2015^67^	Home-based caregivers. N=48. HBC clients N=31	Zambia	Qualitative Thematic analysis	Caregivers spend less time on hands-on physical care and support in the household. Increased clients’ access and adherence to ART^d^.
Shahid 2016^71^	Spouses of HIV-positive men. N=2400	Pakistan	Cross-sectional	HBC^e^ was an effective way of expanding access and identifying cases of undiagnosed HIV
Pokhrel 2018^74^	PLWH. N=682 participants	Nepal	RCT- follow up 6 months	Significant positive effects in reducing depressive symptoms, anxiety, stress, substance use and non-adherence
Bui 2018^70^	PLWH. N=180 participants	Viet Nam	Case control	HBC^e^ was significantly associated with higher self-perceptions of quality of life
Tun 2019^69^	HIV-positive female sex workers. N = 509	Tanzania	RCT-follow up 6 months	Adherence to ART^d^ over standard facility-based ART^d^ programs.

a People living with HIV /AIDS

b Randomized Controlled Trial

c Voluntary Counseling and Testing

d Antiretroviral Therapy; eHome-Based Care

e Home-Based Care

f The 12-Item Short Form Health Survey

g Prevention of Mother-to-Child Transmission

**6.1. Benefits for the patients:** Studies pertaining to benefits of HBC for the patients could be classified into eight categories of patients’ satisfaction, expanding access to services, quality of life, biochemical outcomes, adherence to ART, prevention of mother-to-child transmission, social outcomes, and psychological outcomes. 

**6.1.1. Patients’ satisfaction:** Three studies reported evidence on the use of HBC to increase the patient satisfaction. ^45 , 58 , 68^ A survey conducted in USA reported that clients were very satisfied with HBC delivery. ^45^

**6.1.2. Expanding access to services:** Four studies confirmed the improvement of access to services with HBC in patients. ^67 , 68 , 71 , 77^ In a qualitative study in Mozambique, it is concluded that home care helps to enhance access to HIV and AIDS care in patients. ^67^

**6.1.3 Quality of life:** Nine studies reported evidence on the use of HBC in quality of life. ^32 , 44 , 46 , 49 , 50 , 54 , 62 , 70 , 76^ Using the SF-12 instrument, a study showed HBC interventions have led to a significant improvement in the quality of life associated with health in the psychological dimension. However, this improvement was not observed in the physical dimension in PLWH. ^44^

**6.1.4. Biochemical outcome:** Thirteen studies provided evidence of HBC viral load ^27 , 30 , 33 , 34 , 37 , 38 , 41 - 43 , 53 , 56 , 63 , 78^ and seven studies of CD4 counts. ^27 , 28 , 37 , 51 , 53 , 56 , 63^ An RCT study (2006) reported that no significant difference was seen in lipid levels, blood pressure, or abdominal visceral fat between the groups. ^52^ A randomized control trial in USA examined the effect of an adherence intervention which included social and educational components and reported no signiﬁcant change for the intervention and control groups in terms of the median CD4 counts and viral load. ^53^

**6.1.5. Adherence to Antiretroviral Therapy:** The results of nine studies indicated the increase of compliance with antiretroviral treatments in PLWH. ^29 , 41 , 51 , 62 , 63 , 65 , 67 , 69 , 74^ An RCT reported that the intensive home based nursing intervention signiﬁcantly improved the self-reported adherence to ART. ^51^

**6.1.6. Prevention of mother-to-child transmission:** A survey was conducted in Kenya and its results showed that HBC led to further reduction in vertical transmission of HIV/AIDS. ^57^

**6.1.7. Social outcomes:** The results of studies in USA ^48 , 50^ and Africa ^39 , 50 , 55 , 64 , 75^ based on HBC reported positive social outcomes including family or community support and possibility of overcoming the stigma and reducing discrimination. A study (1998) (n=57) provided information, communication and decision support via a computer in the homes of people living with AIDS and compared it with printed brochures and monthly telephone calls, but did not observe any signiﬁcant difference between groups in terms of health status, decision making conﬁdence and skill. There was a reduction in social isolation in the case of controlling for depression. ^48^

**6.1.8. Psychological outcomes:** Eight studies investigated the effects of HBC on psychological status. ^44 , 47 , 50 , 54 , 62 - 64 , 76^ A study reported the psychosocial impact of home visits carried out by three registered nurses in the homes of African-American women with HIV. After six months, no statistically significant difference was found for depressive symptoms and mood. ^50^

**6.2. Benefits for the families:** Studies related to the benefits of HBC for the family could be classified into three categories of promoting family members’ participation in care, promoting family members’ awareness and providing voluntary counseling and testing (VCT) in the family.

**6.2.1. Promoting family members’ participation in care:** HBC leads to the strengthening of family attachment, ^22 , 60 , 65^ and assists the family to accept the patient’s condition and provide necessary care for patients. ^61^ Also, by reducing medical costs and other care services, ^72^ the family can meet PLWH’s physical, mental and spiritual needs. ^22^

**6.2.2. Promoting family members’ awareness:** HBC helps to properly understand and correct misconceptions about the prevention and treatment of HIV/AIDS in the community. Also, it leads to sustainability in providing care services and improving community practice in providing supportive care. ^22^ According to studies, HBC provides an opportunity for family members to raise awareness about HIV/AID. ^60 , 61^

**6.2.3. Provision of VCT in the family:** HBC could improve VCT delivery to household members of PLWH ^40 , 66^ and identifying undiagnosed cases. ^40 , 71^ Testing household members increases the family diagnosis of HIV infection. In a cross-sectional study in Uganda, of the 176 HIV-positive family members tested, 74% had HIV infection and had not been tested previously. ^40^

**6.3. Benefits for the healthcare systems:** Studies focused on the benefits of HBC for the healthcare system which could be classified into three categories of HBC costs, workload at health centers and time spent on services.

**6.3.1. Health care costs:** Understanding HBC costs is critical for achieving efficacy and health-related outcomes of PLWH for organizations providing care. ^23^ Six studies have evidence about costs of HBC. ^29 , 34 , 36 , 64 , 72 , 73^ Five studies showed that costs of HBC were comparatively favorable to other delivery care. ^29 , 36 , 64 , 72 , 73^ According to a study which compared two home and hospital care models over a period of two years, the results showed that patients in the HBC group had fewer costs than hospital care. ^29^ Also, one study concluded that HBC costs were more cost-effective than health centers-based care in Uganda’s rural areas. ^36^

**6.3.2. Workload at health centers:** Positive outcomes of HBC services in the community include preventing the disease and its complications as a result of the decrease in hospitalization and the need for hospital care. ^73^ This process based on a mixed method study in Uganda will decrease the workload of health centers by reducing the demand for hospital beds and improving the effective use of them. ^31^

**6.3.3. Time spent on services:** Four studies have noted the effects of HBC on the time spent. ^35 , 47 , 49 , 67^ A study in Zambia reported that caregivers spent less time on hands-on physical care and support in the household. ^67^ Based on a study reported, there were no signiﬁcant changes in the number of outpatient visits but a reduced time was spent with providers as well as a signiﬁcantly reduced probability of admission. ^49^

**6.4: Challenges in HBC programs:** The study identifies the problems that the HBC program encounters. Some of the main challenges that HBC programs face based on guidelines include gender inequality and the feminization of care, non-disclosure of disease and trust in service providers, ^12^ as well as the significant health and safety risks, including exposure to infection in a threatened environment, and absence of knowledge and enough health care providers. ^23^ Patient-related stigma, child care methods, confidentiality, high illiteracy rates in target groups, lack of competence and awareness in HBC approach, economic crisis, limited resources and sustainability, human resources, technical skills, reference systems failure, organizational and logistic resources and co-infections. It is also mentioned in the following sections, geographical distributions of patients, strategies for care of providers, lack of community participation, community cultural and religious issues and compromised care in cases where limited resources, attitude, beliefs, values, and misinformation about HIV, the ability of caregivers to provide effective and respectful care of PLWH and their families of other HBC challenges. ^22 , 23^

## DISCUSSION

The results indicate that there is thorough evidence that HBC interventions could impact on morbidity in PLWH and is beneficial for both families and healthcare systems. This approach, by establishing a safe and friendly environment, leads to stabilization of people with chronic illnesses. ^79^ It can also help to create opportunities for implementing new policies in low- and middle-income countries. ^80^ Poverty of patients can directly affect their participation in care, ^81^ because individuals do not have adequate financial resources to attend health centers, such as transportation costs. They also face problems such as waiting time and lost salary due to taking leave from work for going to health centers. ^82^ Additionally, other factors, such as concern about the confidentiality of information and inappropriate hours of referral to clinic, are barriers to get services. ^82^ In contrast, providing HBC helps to overcome some of the barriers in PLWH and their families and decrease the burden on healthcare delivery centers. HBC has also led to an increase in men’s participation in care as well as the elimination of poverty in patients. ^83^

A qualitative study conducted in Iran, about the views of experts on the benefits of HBC, reported that this approach has characteristics of a complete care that includes continuity, quality, availability, cost-effectiveness, and comprehensiveness. However, some of these characteristics may not be available in hospital-based care. Also, it has emphasized the necessity of the establishment and development of this approach in Iran. ^84^

Results of a cross-sectional community-based study in Ethiopia also showed that 92.8% of people believed that home is the best place to care for PLWH compared to hospitals. ^85^ Africa is one of the areas in the world that has high cases of HIV/AIDS. The health sectors, therefore, cannot deal with the number of HIV patients, who require to be admitted in the hospitals. This approach leads to the patients’ stay at home and their families have to take care of them. Most countries in the Southern Africa have adopted community mobilization as a strategic intervention for continuing HBC program. ^86^ The African communities treat HIV/AIDS as stigma and most African people prefer to be cared in their own homes instead of being treated in Hospice Centers or Home-based care centers. ^6^

HHBC has an extensive application to empower communities and families to meet the physical, mental and spiritual needs of PLWH. ^87^ This review had similar findings to a systematic review examining interventions for enhancing adherence to ART, which concluded that nurse-led home-based strategies are effective in HIV/AIDS. ^88^ Despite numerous evidence on the benefits of HBC, there are some studies that have not verified this approach. A clinical trial conducted in Uganda compared clinical outcomes, costs, and adherence to antiretroviral treatments based on HBC and facility-based care. The results showed that there is no difference between the two models in viral load , mortality, CD4 counts, and compliance to treatment, while the costs of providing services are similar for both models. ^34^ Substantial differences between studies may be due to the patients’ clinical status, the setting of study, demographic characteristics of the patients, and resources available across different studies. 

In one study, it has been reported that as benefits of HBC, it should be noted that family members of patients have a negative attitude towards AIDS, which leads to isolation of patients. ^9^ Although knowledge about AIDS is important, the attitudes towards patients must also be positive, as it will increase their behavioral changes. Our study showed that HBC programs with the participation of families could spread knowledge and awareness of HIV and AIDS and improve the attitude of household members of patients. It can serve as an entry point for HIV/AIDS education and prevention at the family and community levels. Also, generalized home-based HIV testing and counseling represents a remarkable opportunity to test the couples, children, and families in order to increase early detection of HIV-positive cases and identification of the first time testers. ^89^

The use of HBC, comparatively, is said to be more cost-effective than other delivery strategies. ^29 , 36 , 64 , 72 , 73^ Indeed, increasing the costs of hospital stay and being away from the family and hospital-acquired complications have caused the HBC services to be more attractive to patients, especially in chronic diseases, which is expected not to be improved in the near future^9090^ These costs are mainly focused on anti-retroviral drugs and hospitalization, ^91^ while providing HBC is of particular importance for patients who are under antiretroviral treatment and can lead to a reduction in the cost of treatment at the individual and social levels. ^80^ It is anticipated that if the governments would recognize the role of the HBC services and, therefore, employ them on a permanent basis as the integration of HBC in the era of HIV, it is expected that the economic and human resource pressures would be reduced, which is itself a burden to the health care systems, especially hospitals. ^92^ In contrast with the benefit of HBC to reduce the time spent, a cohort study in Uganda reported that HBC model has higher waiting times than doctor-based care models because it takes about twice as much time to care for patients. ^35^ This is in contrast with task-shifting models in USA and Zambia that showed a decrease in the time spent by caregivers. ^49 , 67^

In order to better understand the costs of treatment in these patients, it should be noted that accompanying illnesses are common among PLWH that leads to a significant increase in treatment costs through out-of-pocket payments and medical insurances. ^93^ In many developing countries, this type of approach can be funded by the government through public taxations, community-based social insurance schemes, or income from public health services. CSOs (Civil Society Organizations)/NGOs should also make a proper contribution to the budget. In these cases, community-based insurance plans might be a good option.12 Nevertheless, despite the literature review that has shown the benefits of HBC, its implementation is associated with some challenges for organizations, caregivers and patients. ^94^ These challenges might be different in various countries across the world depending on their culture and value system.

A qualitative study conducted in 2016 addressed the obstacles to establishing HBC approach in Iran. The main obstacles included: 1) treatment-based approaches in the Iranian health care system, 2) cultural issues, and 3) lack of adequate infrastructures. ^19^ Some strategies have been provided to overcome these barriers in some guidelines; for instance, in National Guideline for Community and Home-Based Care of Pakistan, it has been mentioned that governments, at first, must review the challenges associated with the issue. Then, they should start to educate, prevent, and care for people living with HIV/AIDS in their community. ^12^ It has been suggested that, in order to establish this approach, the governments should initially approve the issue of care as a priority requirement for individuals, encourage the gender approach to care, and ensure that the HBC staff know about their profession through different channels and have the necessary credit and support. ^12^ Also, a system should be established for recording, quality control measures, job security and better management of HBC plans. ^12 , 22^

Also, implementation of this approach requires the establishment of standards such as: providing care (basic physical, psychological and social care of patients and their affected individuals), continuity of care (access, awareness of resource, community coordination, finding and managing cases and recording their history), training (developing appropriate curricula, providing education to reduce stigma, engaging the media, and evaluating education),as well as supplies and equipments (appropriate selection of care team locations, provision of care packages at home, management, monitoring and maintenance of equipments). Also human resources (recruitment, supervision and coordination of the service provider team, staff retention), financing (budget management, technical support, encouraging volunteers, collecting resources, management of out-of-pocket payments, and free services), as well as monitoring and evaluation (evaluation, quality assurance, quality care indicators survey, monitoring, informal and formal assessment, and flexibility) are important issues. ^12 , 22^

Our study had several strengths. This study was conducted in different contexts with different types of study designs and without limitation of publication year, which provided a broad overview of the benefits and challenges of HBC. Besides, our study has also included all components related to the HBC intervention. As a limitation of our study, no research was found on HBC in PLWH in Iran, so that we did not have sufficient resources about its necessity in our country in the context of HIV. Also, quality assessment of the studies was not conducted in the current study. Within the framework of this research, we reviewed the studies published in English and Persian languages and, therefore, may have omitted relevant articles published in other languages. In order to realize the greater advantages of HBC, we recommend that some quantitative studies should be conducted to further investigate the topic. Also, qualitative studies could help to understand the challenges and facilities needed to deploy this approach in the context of HIV.

## CONCLUSION

In summary, our study found that scheduled HBC is an effective, acceptable and feasible strategy and plays a key role in the care of PLWH, despite its enormous challenges. Therefore, the idea of developing an alternative approach for providing healthcare of PLWH at home is inevitable for Iran’s sociocultural and economic conditions. Extending this approach can eliminate the constraints on the financial, human, and physical infrastructures of the health system, which leads to the lack of regular referral of patients and their affected relatives to get care. Along with this, it might lead to the promotion of community participation, which is one of the important and not well-implemented principles of primary health care in Iran. In this regard, by providing the infrastructures needed to implement HBC, a cost-effective and satisfactory program can be concluded as an achievement in the healthcare system towards fighting against HIV/AIDS. Also, considering the challenges of HBC, the emerging evidence suggests the planning, implementation and sustainability, as well as budget allocation to HBC for PLWH in policy making agenda setting in Iran.

## References

[1] Joint United Nations Programme on HIV/AIDS (UNAIDS) Fact sheet - world AIDS day. Geneva: Joint United Nations Programme on HIV/AIDS (UNAIDS); 2019. [cited 19 February 2019]. http://www.unaids.org/sites/default/files/media_asset/UNAIDS_FactSheet_en.pdf.

[2] Selvaraj K, Kumar A, Chawla S ( 2017). Are partners of HIV-infected people being tested for HIV? A mixed-methods research from Gujarat, India. Public Health Action.

[3] larki M, Bahri N, Moghri J, Latifnejad Rudsari R ( 2020). Living with Discordance: A Qualitative Description of the Challenges Faced by HIV Negative Married Women. International Journal of Community Bassed Nursing and Midwifery.

[4] Khajehkazemi R, Sadeghirad B, Karamouzian M, Fallah M-S, Mehrolhassani M-H, Dehnavieh R ( 2013). The projection of burden of disease in Islamic Republic of Iran to 2025. PloS one.

[5] Kodama S, Horikawa C, Fujihara K ( 2012). Comparisons of the strength of associations with future type 2 diabetes risk among anthropometric obesity indicators, including waist-to-height ratio: a meta-analysis. American Journal of Epidemiology.

[6] Akintola O A gendered analysis of the burden of care on family and volunteer caregivers in Uganda and South Africa. South Africa: Health Economics and HIV/AIDS Research Division (HEARD), University of KwaZulu-Natal; 2004.

[7] Uys LR, Cameron S Home-based HIV/AIDS care. 1st ed. UK: Oxford University Press; 2003.

[8] Osafo J, Knizek BL, Mugisha J, Kinyanda E ( 2017). The experiences of caregivers of children living with HIV and AIDS in Uganda: a qualitative study. Globalization and Health.

[9] Nyaphisi MB, Obioha EE ( 2015). Challenges of HIV and AIDS-related community home-based health care delivery system in Roma Valley, Lesotho. Anthropological Notebooks.

[10] Hove-Musekwa SD, Nyabadza F, Mambili-Mamboundou H ( 2014). Cost-effectiveness analysis of hospitalization and home-based care strategies for people living with HIV/AIDS: the case of Zimbabwe. International Scholarly Research Notices.

[11] Hove-Musekwa S, Nyabadza F, Mambili-Mamboundou H ( 2011). Modelling hospitalization, home-based care, and individual withdrawal for people living with HIV/AIDS in high prevalence settings. Bulletin of Mathematical Biology.

[12] National AIDS Control Programme National Guidelines for Community and Home Based Care. Tanzania: National AIDS Control Programme; 2015.

[13] Iacono SL, Allen E The cooperative model for the delivery of home based care services for people living with HIV. Geneva: International Labour Organization; 2011.

[14] Malale K ( 2011). Home Based Care for People Living with HIV/AIDS; Assessment of Knowledge, Attitude and Practice among Family Care Givers at Ukonga Ward in Ilala District. Dar Es Salaam Medical Students’ Journal.

[15] Amoran OE, Ogunsola EO, Salako AO, Alausa OO ( 2012). HIV/AIDS related home based care practices among primary health care workers in Ogun state, Nigeria. BMC Health Services Research.

[16] Kronenfeld JJ (1982). Organisation of ambulatory care by consumers. Sociology of Health & Illness.

[17] Smith JA, Sharma M, Levin C ( 2015). Cost-effectiveness of community-based strategies to strengthen the continuum of HIV care in rural South Africa: a health economic modelling analysis. The Lancet HIV.

[18] Lotfi Fatemi N, Moonaghi HK, Heydari A ( 2019). Perceived Challenges Faced by Nurses in Home Health Care Setting: A Qualitative Study. International Journal of Community Based Nursing and Midwifery.

[19] Heydari H, Shahsavari H, Hazini A, Nikbakht Nasrabadi A ( 2016). Exploring the barriers of home care services in Iran: A qualitative study. Scientifica.

[20] Shahsavari H, Nikbakht Nasrabadi A, Almasian M ( 2018). Exploration of the administrative aspects of the delivery of home health care services: a qualitative study. Asia Pacific family medicine.

[21] World Health Organization Community home-based care in resource-limited settings: a framework for action. Geneva:World Health Organization; 2002.

[22] The United Republic of Tanzania, Ministry of Health Natinal guidlines for the clinical managment of HIV and AIDS. Tanzania: The United Republic of Tanzania, Ministry of Health; 2005.

[23] His N, Musau S, Chanfreau C HIV/AIDS Home-Based Care Costing Guidelines. US: United States Agency for International Development; 2005.

[24] Joint United Nations Programme on HIV/AIDS Reducing HIV stigma and discrimination: a critical part of national AIDS programmes, A resource for national stakeholders in the HIV response. Geneva: Joint United Nations Programme on HIV/AIDS; 2007.

[25] World Health Organization  Planning, implementing and monitoring home-based HIV testing and counselling: a practical handbook for Sub-Saharan Africa: World Health Organization; 2012.

[26] Moher D, Shamseer L, Clarke M ( 2015). Preferred reporting items for systematic review and meta-analysis protocols (PRISMA-P) 2015 statement. Systematic Reviews.

[27] Mermin J, Lule J, Ekwaru JP ( 2004). Effect of co-trimoxazole prophylaxis on morbidity, mortality, CD4-cell count, and viral load in HIV infection in rural Uganda. The Lancet.

[28] Mermin J, Ekwaru JP, Were W ( 2011). Utility of routine viral load, CD4 cell count, and clinical monitoring among adults with HIV receiving antiretroviral therapy in Uganda: randomised trial. BMJ.

[29] Kipp W, Konde-Lule J, Rubaale T ( 2011). Comparing antiretroviral treatment outcomes between a prospective community-based and hospital-based cohort of HIV patients in rural Uganda. BMC International Health and Human Rights.

[30] Kipp W, Konde-Lule J, Saunders LD ( 2012). Antiretroviral treatment for HIV in rural Uganda: two-year treatment outcomes of a prospective health centre/community-based and hospital-based cohort. PloS One.

[31] Arem H, Nakyanjo N, Kagaayi J ( 2011). Peer health workers and AIDS care in Rakai, Uganda: a mixed methods operations research evaluation of a cluster-randomized trial. AIDS Patient Care and STDs.

[32] Alibhai A, J Martin L, Kipp W ( 2010). Quality of life of HIV patients in a rural area of western Uganda: impact of a community-based antiretroviral treatment program. Current HIV Research.

[33] Kipp W, Konde-Lule J, Saunders LD ( 2010). Results of a community-based antiretroviral treatment program for HIV-1 infection in Western Uganda. Current HIV Research.

[34] Jaffar S, Amuron B, Foster S ( 2009). Rates of virological failure in patients treated in a home-based versus a facility-based HIV-care model in Jinja, southeast Uganda: a cluster-randomised equivalence trial. The Lancet.

[35] Wanyenze RK, Wagner G, Alamo S ( 2010). Evaluation of the efficiency of patient flow at three HIV clinics in Uganda. AIDS Patient Care STDS.

[36] Marseille E, Kahn JG, Pitter C ( 2009). The cost effectiveness of home-based provision of antiretroviral therapy in rural Uganda. Applied Health Economics and Health Policy.

[37] Mermin J, Were W, Ekwaru JP ( 2008). Mortality in HIV-infected Ugandan adults receiving antiretroviral treatment and survival of their HIV-uninfected children: a prospective cohort study. The Lancet.

[38] Amuron B, Coutinho A, Grosskurth H ( 2007). A cluster-randomised trial to compare home-based with health facility-based antiretroviral treatment in Uganda: study design and baseline findings. The Open AIDS Journal.

[39] Apondi R, Bunnell R, Awor A ( 2007). Home-based antiretroviral care is associated with positive social outcomes in a prospective cohort in Uganda. JAIDS Journal of Acquired Immune Deficiency Syndromes.

[40] Were WA, Mermin JH, Wamai N ( 2006). Undiagnosed HIV infection and couple HIV discordance among household members of HIV-infected people receiving antiretroviral therapy in Uganda. Journal of Acquired Immune Deficiency Syndromes.

[41] Weidle PJ, Wamai N, Solberg P ( 2006). Adherence to antiretroviral therapy in a home-based AIDS care programme in rural Uganda. The Lancet.

[42] Bunnell R, Ekwaru JP, Solberg P ( 2006). Changes in sexual behavior and risk of HIV transmission after antiretroviral therapy and prevention interventions in rural Uganda. AIDS.

[43] Amuron B, Levin J, Birunghi J ( 2011). Mortality in an antiretroviral therapy programme in Jinja, south-east Uganda: a prospective cohort study. AIDS Research and Therapy.

[44] Blank MB, Hennessy M, Eisenberg MM ( 2014). Increasing quality of life and reducing HIV burden: the PATH+ intervention. AIDS and Behavior.

[45] Foley ME, Fahs MC, Eisenhandler J, Hyer K ( 1995). Satisfaction with home healthcare services for clients with HIV: preliminary findings. Journal of the Association of Nurses in AIDS Care.

[46] Nickel JT, Salsberry PJ, Caswell RJ ( 1996). Quality of life in nurse case management of persons with AIDS receiving home care. Research in Nursing & Health.

[47] Cherin DA, Huba G, Brief DE, Melchior LA ( 1998). Evaluation of the transprofessional model of home health care for HIV/AIDS. Home Health Care Services Quarterly.

[48] Flatley-Brennan P ( 1998). Computer network home care demonstration: a randomized trial in persons living with AIDS. Computers in Biology and Medicine.

[49] Gustafson DH, Hawkins R, Boberg E ( 1999). Impact of a patient-centered, computer-based health information/support system. American Journal of Preventive Medicine.

[50] Miles MS, Holditch-Davis D, Eron J ( 2003). An HIV self-care symptom management intervention for African American mothers. Nursing Research.

[51] Berrien VM, Salazar JC, Reynolds E, Mckay K ( 2004). Adherence to antiretroviral therapy in HIV-infected pediatric patients improves with home-based intensive nursing intervention. AIDS Patient Care and STDs.

[52] Dolan SE, Frontera W, Librizzi J ( 2006). Effects of a supervised home-based aerobic and progressive resistance training regimen in women infected with human immunodeficiency virus: a randomized trial. Archives of Internal Medicine.

[53] Williams AB, Fennie KP, Bova CA ( 2006). Home visits to improve adherence to highly active antiretroviral therapy: a randomized controlled trial. JAIDS Journal of Acquired Immune Deficiency Syndromes.

[54] Hanrahan NP, Wu E, Kelly D ( 2011). Randomized clinical trial of the effectiveness of a home-based advanced practice psychiatric nurse intervention: outcomes for individuals with serious mental illness and HIV. Nursing Research and Practice.

[55] Wools-Kaloustian KK, Sidle JE, Selke HM ( 2009). A model for extending antiretroviral care beyond the rural health centre. Journal of the International AIDS Society.

[56] Selke HM, Kimaiyo S, Sidle JE ( 2010). Task-shifting of antiretroviral delivery from health care workers to persons living with HIV/AIDS: clinical outcomes of a community-based program in Kenya. Journal of Acquired Immune Deficiency Syndromes.

[57] Kohler PK, Okanda J, Kinuthia J ( 2014). Community-based evaluation of PMTCT uptake in Nyanza Province, Kenya. PLoS One.

[58] McCann K ( 1991). The work of a specialist AIDS home support team: the views and experiences of patients using the service. Journal of Advanced Nursing.

[59] Butters E, Higginson I, George R ( 1991). Assessing the symptoms, anxiety and practical needs of HIV/AIDS patients receiving palliative care. Quality of Life Research.

[60] Butters E, Higginson I ( 1995). Two HIV/AIDS community support teams: patient characteristics, problems at referral and during the last 6 weeks of life. Aids Care.

[61] Koffman J, Higginson I, Naysmith A ( 1996). Hospice at home--a new service for patients with advanced HIV/AIDS: a pilot evaluation of referrals and outcomes. British Journal of General Practice.

[62] Wang H, Zhou J, Huang L ( 2010). Effects of nurse-delivered home visits combined with telephone calls on medication adherence and quality of life in HIV-infected heroin users in Hunan of China. Journal of Clinical Nursing.

[63] Williams AB, Wang H, Li X ( 2014). Efficacy of an evidence-based ARV adherence intervention in China. AIDS Patient Care and STDs.

[64] Uys L ( 2001). Evaluation of the integrated community based home care model. Curationis.

[65] Wouters E, van Loon F, van Rensburg D, Meulemans H ( 2009). Community support and disclosure of HIV serostatus to family members by public-sector antiretroviral treatment patients in the Free State Province of South Africa. AIDS Patient Care STDS.

[66] Fylkesnes K, Siziya S ( 2004). A randomized trial on acceptability of voluntary HIV counselling and testing. Tropical Medicine & International Health.

[67] Cataldo F, Kielmann K, Kielmann T ( 2015). ‘Deep down in their heart, they wish they could be given some incentives’: a qualitative study on the changing roles and relations of care among home-based caregivers in Zambia. BMC Health Services Research.

[68] Decroo T, Telfer B, Biot M ( 2011). Distribution of antiretroviral treatment through self-forming groups of patients in Tete Province, Mozambique. JAIDS Journal of Acquired Immune Deficiency Syndromes.

[69] Tun W, Apicella L, Casalini C ( 2019). Community-Based Antiretroviral Therapy (ART) Delivery for Female Sex Workers in Tanzania: 6-Month ART Initiation and Adherence. AIDS and Behavior.

[70] Bui QTT, Brickley DB, Tieu VTT, Hills NK ( 2018). Home-Based Care and Perceived Quality of Life Among People Living with HIV in Ho Chi Minh City, Viet Nam. AIDS and Behavior.

[71] Shahid S, Majeed MF, Awaan AB ( 2016). Expanding access to HIV testing and counseling and exploring vulnerabilities among spouses of HIV-positive men who inject drugs in Pakistan. Current Opinion in HIV and AIDS.

[72] Tramarin A, Milocchi F, Tolley K ( 1992). An economic evaluation of home-care assistance for AIDS patients: a pilot study in a town in northern Italy. AIDS.

[73] Bunch EH ( 1998). AIDS in Norway: a post hoc evaluation of an AIDS home care project. Journal of Clinical Nursing.

[74] Pokhrel KN, Sharma VD, Pokhrel KG ( 2018). Investigating the impact of a community home-based care on mental health and anti-retroviral therapy adherence in people living with HIV in Nepal: a community intervention study. BMC Infectious Diseases.

[75] Oguntibeju OO, Ndalambo KT, Mokgatle-Nthabu M ( 2011). People living with HIV/Aids and the utilisation of home-based care services. African Journal of Microbiology Research.

[76] Moons M, Kerkstra A, Biewenga T ( 1994). Specialized home care for patients with AIDS: an experiment in Rotterdam, The Netherlands. Journal of Advanced Nursing.

[77] Yoder PS, Katahoire AR, Kyaddondo D Home-based HIV testing and counselling in a survey context in Uganda. US: United States Agency for International Development; 2006.

[78] Moore DM, Yiannoutsos CT, Musick BS ( 2011). Determinants of early and late mortality among HIV-infected individuals receiving home-based antiretroviral therapy in rural Uganda. Journal of Acquired Immune Deficiency Syndromes.

[79] Health Quality Ontario ( 2013). In-home care for optimizing chronic disease management in the community: an evidence-based analysis. Ontario health technology assessment series.

[80] Wringe A, Cataldo F, Stevenson N, Fakoya A ( 2010). Delivering comprehensive home-based care programmes for HIV: a review of lessons learned and challenges ahead in the era of antiretroviral therapy. Health Policy and Planning.

[81] Tsai AC, Bangsberg DR, Weiser SD ( 2013). Harnessing poverty alleviation to reduce the stigma of HIV in Sub-Saharan Africa. PLoS Medicine.

[82] Leichliter JS, Paz-Bailey G, Friedman AL ( 2011). ‘Clinics aren’t meant for men’: Sexual health care access and seeking behaviours among men in Gauteng province, South Africa. Journal of Social Aspects of HIV/AIDS.

[83] Sharma M, Barnabas RV, Celum C ( 2017). Community-based strategies to strengthen men’s engagement in the HIV care cascade in sub-Saharan Africa. PLoS Medicine.

[84] Barati A, Janati A, Tourani S (2010). Iranian professional’s perception about advantages of developing home health care system in Iran.

[85] Tibebu B, G/Mariam A, Belachew T ( 2007). Knowledge, attitude and practice of home-based care for HIV/AIDS patients by their family/caregivers at Jimma Town. Ethiopian Medical Journal.

[86] United Nations Programme on HIV/AIDS (UNAIDS) Report on the global AIDS epidemic. Geneva: United Nations Programme on HIV/AIDS (UNAIDS); 2008.

[87] Va  Dyk AC HIVAIDS care and counselling: a multidisciplinary approach. 4th ed. South Africa: Pearson South Africa; 2010.

[88] Mbuagbaw L, Sivaramalingam B, Navarro T ( 2015). Interventions for enhancing adherence to antiretroviral therapy (ART): a systematic review of high quality studies. AIDS Patient Care and STDs.

[89] Labhardt ND, Motlomelo M, Cerutti B ( 2014). Home-based versus mobile clinic HIV testing and counseling in rural Lesotho: a cluster-randomized trial. PLoS Medicine.

[90] Zingmond DS, Arfer KB, Gildner JL, Leibowitz AA ( 2017). The cost of comorbidities in treatment for HIV/AIDS in California. PloS One.

[91] Kates J Medicaid and HIV: a national analysis. California (US): Kaiser Family Foundation; 2011.

[92] Makoae M, Jubber K ( 2008). Confidentiality or continuity? Family caregivers\’experiences with care for HIV/AIDS patients in homebased care in Lesotho. Journal of Social Aspects of HIV/AIDS.

[93] Meyer N, Gallant J, Hsue P, Song X Comorbidities of Patients with Human Immunodeficiency Virus (HIV) in the USA-a Longitudinal Analysis of Prevalent HIV Patients Over 11 Years. Paper presented at: 55th Interscience Conference on Antimicrobial Agents and Chemotherapy; 2015 September 5-9; San Diego, California.

[94] Kangethe S ( 2009). Critical coping challenges facing caregivers of persons living with HIV/AIDS and other terminally III persons: The case of Kanye Care Program, Botswana. Indian Journal of Palliative Care.

